# Emerging Pleiotropic Mechanisms Underlying Aluminum Resistance and Phosphorus Acquisition on Acidic Soils

**DOI:** 10.3389/fpls.2018.01420

**Published:** 2018-09-26

**Authors:** Jurandir V. Magalhaes, Miguel A. Piñeros, Laiane S. Maciel, Leon V. Kochian

**Affiliations:** ^1^Embrapa Maize and Sorghum, Sete Lagoas, Brazil; ^2^Departamento de Biologia Geral, Universidade Federal de Minas Gerais, Belo Horizonte, Brazil; ^3^Robert W. Holley Center for Agriculture and Health, USDA-ARS, Cornell University, Ithaca, NY, United States; ^4^Global Institute for Food Security, University of Saskatchewan, Saskatoon, SK, Canada

**Keywords:** abiotic stress resistance, transporters, plant breeding, pleiotropy, aluminum tolerance, phosphorus acquisition, phosphorus efficiency

## Abstract

Aluminum (Al) toxicity on acidic soils significantly damages plant roots and inhibits root growth. Hence, crops intoxicated by Al become more sensitive to drought stress and mineral nutrient deficiencies, particularly phosphorus (P) deficiency, which is highly unavailable on tropical soils. Advances in our understanding of the physiological and genetic mechanisms that govern plant Al resistance have led to the identification of Al resistance genes, both in model systems and in crop species. It has long been known that Al resistance has a beneficial effect on crop adaptation to acidic soils. This positive effect happens because the root systems of Al resistant plants show better development in the presence of soil ionic Al^3+^ and are, consequently, more efficient in absorbing sub-soil water and mineral nutrients. This effect of Al resistance on crop production, by itself, warrants intensified efforts to develop and implement, on a breeding scale, modern selection strategies to profit from the knowledge of the molecular determinants of plant Al resistance. Recent studies now suggest that Al resistance can exert pleiotropic effects on P acquisition, potentially expanding the role of Al resistance on crop adaptation to acidic soils. This appears to occur *via* both organic acid (OA)- and non-OA transporters governing a joint, iron-dependent interplay between Al resistance and enhanced P uptake, *via* changes in root system architecture. Current research suggests this interplay to be part of a P stress response, suggesting that this mechanism could have evolved in crop species to improve adaptation to acidic soils. Should this pleiotropism prove functional in crop species grown on acidic soils, molecular breeding based on Al resistance genes may have a much broader impact on crop performance than previously anticipated. To explore this possibility, here we review the components of this putative effect of Al resistance genes on P stress responses and P nutrition to provide the foundation necessary to discuss the recent evidence suggesting pleiotropy as a genetic linkage between Al resistance and P efficiency. We conclude by exploring what may be needed to enhance the utilization of Al resistance genes to improve crop production on acidic soils.

## Introduction

Acidic soils are globally widespread, extending to more than half of the world arable lands (von Uexküll and Mutert, 1995). These low-pH soils, which are commonly found in tropical and subtropical regions, include areas where food production needs to be increased to cope with a continuously growing population (Godfray et al., 2010). For example, there have been a number of studies in the literature addressing the extent of Al-toxic acidic soils in Africa, with approximately 25% of the soils being highly acidic (FAO and ITPS, 2015; Tully et al., 2015). Two of the major constraints for crop production on acidic soils, including those in Sub-Saharan Africa (Doumbia et al., 1993, 1998), are aluminum (Al) toxicity and low phosphorus (P) availability.

Aluminum and iron (Fe) oxides, which are enriched in the clay fraction of acidic soils upon intensive weathering of primary minerals (Shaw, 2001), drive both types of abiotic stresses, causing a general coincidental occurrence of Al toxicity and low P availability on tropical soils (Sanches and Salinas, 1981). Phosphorus forms strong, covalent bounds with these oxides, becoming highly unavailable for uptake by the plants (Marschner, 1995; Lynch, 2011), due to restricted P diffusive fluxes from the soil toward the root surface. In addition, P diffusion on highly weathered soils is highly dependent on the soil water content (Novais and Smith, 1999), which varies during the crop season, making P supply to the plant and, consequently, P uptake, highly discontinuous. Under low pH, Al present in aluminosilicates and oxides is released as the rhizotoxic Al^3+^ ion into the soil solution, damaging the root system and inhibiting root growth (Delhaize and Ryan, 1995).

Aluminum resistance has long been associated with overall crop adaptation to acidic soils by indirectly enhancing mineral nutrient uptake and drought resistance (Foy et al., 1993). Accordingly, undamaged, “Al resistant” root systems are more effective in absorbing sub-soil water, and nutrients, particularly those that are highly unavailable on acidic soils, such as P. It is important to note that Al toxicity typically extends to sub-soil layers, where liming is highly ineffective in increasing soil pH, enhancing the deleterious effects of drought stress in reducing crop yields.

The widespread nature of Al toxicity and its global impact has spurred extensive research on the physiological, genetic, and molecular mechanisms that enable crops to withstand Al toxicity on acidic soils. Clearly, impressive progress has been made in the last two decades on the molecular underpinnings of crop Al resistance (reviewed by Kochian et al., 2015). These discoveries led to the isolation of a number of the previously anonymous molecular determinants of Al resistance in loci that had been identified previously *via* genetic mapping in crops such as wheat, barley, rye, sorghum, and maize, as well as in model systems such as in *Arabidopsis thaliana*.

It is reasonable to expect that the identification of the molecular drivers of plant Al resistance can be instrumental in the development of novel strategies for improving crop performance on acidic soils in a more efficient way. Marker-assisted backcross to improve Al resistance based on single major loci has been a feasible approach long before major Al resistance genes were cloned. Beyond that, these genes now offer opportunities for large scale germplasm screening approaches based on functional markers, which can streamline the utilization of large germplasm banks in favor of plant breeding (Tanksley and McCouch, 1997; Hufnagel et al., 2018). Most importantly, it is possible that the value of Al resistance for crop production in the context of the multiple stress scenario on acidic soil regions (Bahia Filho et al., 1997) has been somewhat underappreciated. Some possible reasons for that are the lack of systematic efforts to map Al saturation both in the surface and below ground soils and a rather incomplete quantification of the grain yield effect of known Al resistance genes *in soil*, which is to some extent understandable due to the highly complex chemical nature of acidic soils.

There is now an interesting body of emerging evidence suggesting that Al resistance genes may have an additional, pleiotropic effect on acidic soils, which involves enhancement of P acquisition. In conjunction with the known effect of Al resistance in enhancing water and mineral uptake, by promoting better root growth on acidic soils, this would further justify deliberate efforts to design novel, gene-based molecular breeding strategies aimed at developing cultivars adapted to acidic soil regions. These strategies can help in realizing the great potential there is in expanding the world’s agricultural frontier, by exploring the vast areas under acidic soils in the tropics and subtropics, which show in general a favorable topography for agriculture (Sanches and Salinas, 1981).

Here, our objective is not to review the current available information on plant Al resistance or P efficiency, which is defined here as improved performance in soils with low P availability. For that, readers are directed to many available comprehensive reviews (Delhaize and Ryan, 1995; Kochian, 1995; Ma et al., 2001; Kochian et al., 2004; Delhaize et al., 2007, 2012; López-Arredondo et al., 2014; Eekhout et al., 2017). Our goal here is to explore the emerging connections between Al resistance genes and P deficiency responses that help maintain favorable P nutrition, which happens possibly *via* alterations in root system architecture. We recognize these studies are just emerging and are still found largely in the realm of model species, in this case, Arabidopsis. This makes some of the crop-related implications drawn in this paper somewhat speculative in nature. However, due to the efficacy and breeding potential of common mechanisms underlying two important abiotic stress factors on acidic soils, taking advantage of the convergence of Al resistance and P efficiency *via* pleiotropic genes could have a significant impact in enhancing global food security. In the next section, we will briefly review the components comprising mechanisms that might jointly control Al resistance and P nutrition. We will then explore the emerging, underlying basis for such pleiotropy and will close with a brief discussion of the future directions to further explore Al resistance genes as tools to improve P acquisition and crop performance on acidic soils.

## Overview of Possible Pleiotropic Mechanisms Controlling Both Al Resistance and Root Traits That May Lead to Enhanced Phosphorus Acquisition Under Low P Conditions

### Physiological Basis

The ability of a plant to tolerate low P availability in the soil may be achieved both by internal mechanisms, acting to optimize the way plants internally utilize phosphorus, and by mechanisms to improve phosphorus acquisition from the soil. Mendes et al. (2014) genetically assessed the contribution of those mechanisms in maize grown on a tropical soil with low P availability and found that 80% of the QTLs mapped for P acquisition efficiency co-localized with those for P use efficiency (i.e., the ratio between grain yield and the amount of P supplied to the crop), indicating that the efficiency in acquiring P is the main determinant of P use efficiency in tropical maize. Since P acquisition efficiency achieved *via* changes in root morphology is the physiological basis of possible pleiotropy between Al resistance and better P nutrition, here we will briefly discuss this mechanism. For a broader view of mechanisms possibly contributing to enhanced crop performance under low P, which may involve modulation of P transporters, root system architecture modifications in response to low P, exudation of organic acids (OAs) and phosphatases, and mycorrhizal associations, in addition to internal mechanisms of P efficiency, readers are directed to recent reviews in this area (e.g., López-Arredondo et al., 2014).

Since P is in general highly unavailable on acidic soils, results such as those reported by Mendes et al. (2014) are expected, as enhanced capacity to acquire P is the logical first limiting step for P efficiency. However, other mechanisms have also been shown to exert beneficial effects on crop performance under low P in the field (López-Arredondo et al., 2014). The work by Gamuyao et al. (2012) provided a molecular foundation for the importance of root system architecture on the efficiency with which plants acquire P on soils with low P availability. The rice serine/threonine receptor-like kinase, OsPSTOL1, which is a member of the LRK10L-2 subfamily, was shown to enhance early root growth and grain yield on a P-deficient soil *via* increased P uptake, regulating crown root development (Gamuyao et al., 2012). Subsequently, a low but positive correlation between root surface area assessed in younger plants and grain yield under low P was instrumental in the identification of sorghum homologs of *OsPSTOL1*, designated *SbPSTOL1* genes, that also act to enhance root growth, thereby leading to enhanced P acquisition and grain yield in a sorghum association panel (Hufnagel et al., 2014). Mechanistically, plant P deficiency leads to inhibition of primary root growth due to a shift from an indeterminate to a determinate developmental program, which is caused by reduced cell elongation followed by the loss of meristematic cells in the root apical meristem (RAM) (Sánchez-Calderón et al., 2005). Hence, this release of apical dominance leads to enhanced proliferation of lateral roots, and increased lateral root branching increasing P uptake as observed in maize (Zhu and Lynch, 2004; Postma et al., 2014).

From the physicochemical standpoint, the supply of a nutrient like P from the soil solution toward the root surface *via* a diffusive flow can be modeled by the Fick’s law (Nobel, 1991), which depends on the P concentration gradient generated by the interplay between root P absorption and P in the soil solution. This concentration gradient can thus be thought as the “force” driving diffusion fluxes; as the root system grows into new soil regions still rich in P, the distance through which diffusion occurs is reduced, thus enhancing the diffusive flow (Novais and Smith, 1999), which is also maintained by the uptake process. Finally, we point out that changes in the three-dimensional configuration of the root system, such as proliferation of shallow roots, can also enhance P uptake [for more details on such mechanisms, please see Li et al. (2016) and Lynch (2011)].

### Molecular Basis

#### Malate and Citrate Transporters

Organic acid transport and homeostasis is emerging as a central hub in a network of acidic soil stress responses. The first OA transporters involved in Al resistance were the wheat TaALMT1 and Arabidopsis AtALMT1, both shown to encode plasma membrane anion channel proteins that mediate root tip malate efflux (Sasaki et al., 2004; Hoekenga et al., 2006; Piñeros et al., 2008; Zhang et al., 2008). Although being the founding members of a novel class of plant anion transporters, it is now well established that, as a family, ALMT functions extend well beyond Al resistance, and participate in a variety of other physiological processes, including guard cell regulation, fruit quality, anion homeostasis, seed development, and plant–microbe interactions (Sharma et al., 2016). However, electrophysiological analysis of TaALMT1 and AtALMT1 (i.e., those transporters associated with Al-dependent responses) in heterologous systems has shown a distinct functional feature of these two transporters in that although they have transport activity in the absence of extracellular Al^3+^, this activity is enhanced by extracellular Al^3+^ (Hoekenga et al., 2006; Piñeros et al., 2008). This so-called “Al activation” is analogous to processes occurring in ligand-gated channels, with the agonistic binding of Al^3+^ to the ALMT protein triggering a conformational change that favors its open state, consequently increasing its transport activity and facilitating anion (i.e., malate) flux. Although the molecular determinants involved in the binding of Al^3+^ to the ALMT protein remain unknown, a combination of functional analysis of structurally modified TaALMT1 and AtALMT proteins and phylogenetic studies on ALMTs indicate that several different domains in these two proteins are likely to act together in the Al-mediated enhancement of transport activity (Sasaki et al., 2004; Furuichi et al., 2010; Ligaba et al., 2013). Overall, the Al-dependent enhancement of the transport activity of an anion channel mediating the selective efflux of malate represents an elegant regulatory component of root malate exudation associated with Al exclusion processes.

More recently, a second novel transport substrate and new regulatory mechanisms have been described for the TaALMT1 transporter (Ramesh et al., 2015, 2018). It has generally been assumed that malate efflux is the primary transport function associated with TaALMT1. Recently, it was shown that TaALMT1 also has a high permeability to the non-protein amino acid, gamma-aminobutyric acid (GABA), a zwitterion molecule associated with signaling cascades in plants. GABA is not only transported by TaALMT1 but also modulates the activity of the transporter protein. Similarly, the apoplastic pH and anion composition also appear to regulate TaALMT1 transport activity, such that increased anion concentrations and/or more alkaline apoplastic conditions stimulate transport activity (Ramesh et al., 2015). These functional characteristics provide additional regulatory layers to Al^3+^-mediated regulation of TaALMT1 activity. Consequently, in alkaline environments, enhancement of TaALMT1 activity resulting in both malate and GABA efflux has been suggested by Ramesh et al. (2015) to promote extracellular acidification *via* H^+^ efflux coupled to the efflux of the malate anion, thereby potentially ameliorating and providing tolerance to high pH soils. Verification of such a tolerance mechanism operating in response to alkaline environments, and validation of the tantalizing functional plasticity of TaALMT1 in tolerance to abiotic stresses, awaits further investigation. It should be noted that the initial studies on this topic have not found increased tolerance or malate efflux in plants grown on alkaline soils and hydroponic media simulating alkaline field conditions (Silva et al., 2018).

The second type of Al resistance OA transporters belong to a subgroup of plasma membrane-localized MATE transporters identified from the map-based cloning of the major Al resistance loci in sorghum (*SbMATE*) (Magalhaes et al., 2004, 2007) and barley (*HvAACT1*) (Furukawa et al., 2007; Wang et al., 2007). Functional characterization of SbMATE, HvAACT1, and subsequently identified homologs in Arabidopsis (AtMATE1) (Liu et al., 2009), maize (ZmMATE1) (Maron et al., 2009), wheat (Ryan et al., 2009; Tovkach et al., 2013), rice bean (VuMATE1/2) (Yang et al., 2011; Liu et al., 2018), and rice (OsFRD2/4) (Yokosho et al., 2011, 2016) indicates that this subgroup of MATE transporters mediate citrate transport, and therefore as with ALMTs, these transporters underlie Al-exclusion *via* root tip OA root release. However, it is worthwhile to comment about the common assumption that ALMTs and MATEs are functionally very similar, as this is not the case. The functional analysis of several of the MATE transporters involved in Al resistance has established that, when expressed in heterologous systems, this subgroup of MATE transporters mediates constitutive pH-dependent citrate transport that is not activated by Al^3+^ in *Xenopus oocytes* (Magalhaes et al., 2007; Maron et al., 2009; Yang et al., 2011; Melo et al., 2013; Doshi et al., 2017; Liu et al., 2018), although some exceptions have been also reported both in X. oocytes (Furukawa et al., 2007; Yokosho et al., 2011) and tobacco suspension cells (Yokosho et al., 2016). Electrophysiological analysis indicates that, in the absence of exogenous intracellular citrate, these MATE transporters mediate an electrogenic transport that appears to be due to a large cation influx (H^+^, Na^+^, and/or K^+^). Differences in the OA transport mechanism between ALMTs and MATEs raises interesting questions. Because of the large inwardly directed voltage gradient or membrane potential across the root cell plasma membrane, the efflux of the malate and citrate anions is a thermodynamically passive process. This is consistent with the ALMT transporters functioning as anion channels mediating the passive movement of the malate anion out of the root cell.

On the other hand, the MATE transporters use a thermodynamically active (H^+^-driven) antiport mechanism associated with the passive efflux of citrate^2-^ anions down its outwardly directed electrochemical gradient. One interesting and quite speculative explanation for this is that an alternative substrate, rather than the free citrate^2-^ anion, is the substrate being transported out of the root cells. In the recent publication by Doshi et al. (2017), electrophysiological, radiolabeled, and fluorescence-based transport assays in two heterologous expression systems (oocytes and yeast) demonstrated that SbMATE has a fairly broad substrate recognition, mediating proton and/or sodium-driven efflux of the ^14^C-citrate anion, as well as efflux of the organic monovalent cation, ethidium, but not its divalent analog, propidium.

Consistent with those findings, MATE proteins were found to transport a wide range of organic substrates (Omote et al., 2006), both anionic and cationic (Tanihara et al., 2007), and including ethidium in the case of the first characterized MATE family protein, the bacterial MATE, NorM (Morita et al., 2000). Nevertheless, it was somewhat surprising to the field of MATE researchers when it was discovered that the plant MATEs involved in Al resistance mediate the efflux of the anion, citrate. Thus, the findings in the recent Doshi et al. publication showing that at least SbMATE has a more broad transport substrate recognition allows us to very speculatively propose that SbMATE (and its orthologs) mediate the efflux of a complexed rather than free anionic form of citrate. This alternative could help explain the antiporter nature of these MATE transporters, as Al–citrate complexes, for instance, could actively be removed from the symplasm in a process energized by passive H^+^ influx. Under this scenario, this group of MATE transporters would still mediate an Al resistance response by actively removing and detoxifying Al from the symplasm of root cells (i.e., mediating resistance), rather than mediating a process where Al is prevented from entering the root cell.

#### Malate and Citrate Transporters as Part of a Common Stress-Responsive Hub

Transcription factors including the Cys_2_His_2_-type zinc finger transcription factors OsART1 in rice (Yamaji et al., 2009) and AtSTOP1 and 2 (Sawaki et al., 2009), AtWRKY46 (Ding et al., 2013) in Arabidopsis, and the rice ASR (abscisic acidic, stress, and ripening) 1 and 5 (Arenhart et al., 2013, 2016; Lima et al., 2011), are involved with the regulation of membrane transporter genes. OsART1, an AtSTOP1 ortholog, modulates the expression of a number of membrane transporters involved in rice Al resistance, OsNrat1, OsMGT1, and OsFRDL4 (Xia et al., 2010; Yokosho et al., 2011; Chen et al., 2013). Similarly, AtSTOP1 modulates the expression of membrane transporters associated with Al resistance including *AtALMT1, AtMATE1*, and *AtALS3* (Liu et al., 2009; Sawaki et al., 2009), in response to both Al and H^+^ rhizotoxicity. Recently, as discussed in the next sections, changes in *AtSTOP1* regulation of *AtALMT1* have been shown to constitute a major component of P sensing pathways (Balzergue et al., 2017; Mora-Macías et al., 2017). Likewise, expression of *AtALMT1* is also regulated by other signaling pathways involving reactive oxygen species (ROS) and phytohormones (Daspute et al., 2017). Biotic stresses, such as that caused by infection of shoots by pathogenic *Pseudomonas syringae*, also triggered upregulation of *AtALMT1* expression and increased root malate exudation, which attracts the beneficial rhizobacterium, *Bacillus subtilis*, into the root microbiome and stimulates Arabidopsis immune responses (Rudrappa et al., 2008). Overall, these more recent observations indicate that the regulatory role of *AtSTOP1* on *AtALMT1* expression and associated physiological stress responses extend well beyond the original signaling roles associated with Al and H^+^ stress.

#### Al Resistance Transporters That Do Not Transport Organic Acids: Aluminum-Sensitive 3 (ALS3)

Screening for Arabidopsis mutants with altered responses to Al toxicity led to the identification of mutants with increased sensitivity to Al, within which the recessive Al sensitive mutant, *als3*, showed 80% root growth inhibition by Al compared to 24–38% inhibition in the wild type (Larsen et al., 1996). This Al sensitive response was unrelated to enhanced Al uptake by *als3* plants (Larsen et al., 1997). Subsequently, map-based cloning identified ALS3 as an ABC transporter-like protein that is localized to leaf hydathodes and the phloem, in addition to the root cortex (Larsen et al., 2005). Based on its likely plasma membrane localization, it was suggested that ALS3 functions in an Al-specific manner to move Al away from sensitive tissues, thus providing Al resistance. ABC transporters contain both a nucleotide (ATP)-binding domain and a transmembrane (TM) domain (Rea, 2007). Larsen and colleagues noted that both ALS3 and the homologous putative bacterial metal resistance protein, ybbM, do not possess the ATP binding domain, which is normally needed for ABC transporters to function.

The ABC transporter, *sensitive to Al rhizotoxicity* (AtSTAR1), which possesses only the ATP-binding domain and not the TM domain, was implicated in Al resistance in Arabidopsis (Huang et al., 2010). AtSTAR1 is a homolog of rice OsSTAR1. Huang et al. (2009) showed that OsSTAR1 (which contains the nucleotide-binding domain) forms an ABC complex with OsSTAR2 (which contains the TM domain), which results in an active ABC transporter involved in Al resistance possibly by mediating UDP glucose efflux into the rice root cell wall. The actual mechanism whereby this activated form of glucose may provide Al tolerance still remains to be elucidated. However, Huang and collaborators hypothesize that UDP glucose may be transported by membrane-localized STAR1–STAR2 from the cytosol into vesicles, from which either UDP-glucose or derived glycoside would be released into the apoplast *via* exocytosis across the plasma membrane, and used to mask the sites for Al binding in the cell wall, thus providing Al resistance. In Arabidopsis, Huang et al. (2010) presented findings suggesting that AtSTAR1 may form a complex with ALS3, with ALS3 providing the TM domain enabling the formation of a functional AtSTAR1/ALS3 complex, which may mediate Al efflux from the outer cell layers of the root tip. These findings indicate that Arabidopsis Al resistance is complex, and also include AtALMT1 (Hoekenga et al., 2006) and AtMATE (Liu et al., 2009) providing root Al exclusion *via* root malate and citrate efflux. In addition to ALS3, a number of other putative Al transporters have been identified that could mediate Al resistance. These include OsNrat1, a rice root plasma membrane uptake transporter that ultimately results in Al storage in the root vacuole (Xia et al., 2010), AtNIP1, a root tip plasma membrane aquaporin protein that mediates root Al uptake (as an Al–malate complex) and sequestration (Wang et al., 2017), and another Arabidopsis ABC transporter, ALS1 (Larsen et al., 2007; Nezames et al., 2012).

Research based on suppressor screens have focused on the identification of molecular factors in the form of mutations that could complement the Al-sensitive phenotype of *als3* (Gabrielson et al., 2006). These studies implicated DNA damage as a biochemical target of Al (Rounds and Larsen, 2008; Nezames et al., 2012; Sjogren et al., 2015; Sjogren and Larsen, 2017), which is viewed as a possible venue to enhance crop Al resistance (Eekhout et al., 2017). One component is the cell cycle checkpoint factor, *ALUMINUM TOLERANT2* (*ALT2*), which may recruit members of the machinery involved with the detection and repair of DNA damage elicited by Al toxicity (Nezames et al., 2012). Accordingly, it was proposed that ALT2, and also ataxia telangiectasia-mutated and Rad3-related (ATR), impair the cell cycle and drive quiescent center differentiation in response to DNA damage caused by Al, leading to root growth arrest elicited by Al. It will be very interesting to assess the effect of the molecular factors involved with the biochemical targets of Al toxicity, such as DNA damage, in enhancing crop performance on acidic soils. Genetic manipulation of the underlying factors for Al toxicity is thought to hold potential for increasing global food security on acidic soils (Rounds and Larsen, 2008). Within the realm of natural variation for Al resistance in crop plants, the allelic effects of such factors may prove to be milder compared to that of major Al resistance genes encoding plasma membrane transporters. Nevertheless, exploiting such distinct biochemical pathways in concert, in the context of plant breeding, may offer potential for identifying transgressive segregants that could enhance even further crop perform on acidic soils.

## Possible Pleiotropic Effects Underlying Al Resistance and P Acquisition Efficiency

### SbMATE and TaALMT1 Increase Grain Yield on Al-Toxic and P-Deficient Soils

Overexpression of the wheat Al resistance gene, *TaALMT1*, in transgenic barley under the control of the ubiquitin promoter has been shown to enhance both P uptake and grain production on an acidic, high P-fixing soil (Delhaize et al., 2009). This effect was attributed in large part to the role of TaALMT1 in maintaining root growth under soil acidity, which likely results from Al resistance. However, the observed greater P uptake per unit length in *TaALMT1*-expressing barley lines might also have resulted to some extent from P mobilization from the soil clays by the malate released into the rhizosphere, thus favoring P uptake (Delhaize et al., 2009). When the soil was limed, which substantially reduced Al saturation, grain yield of the transgenic and non-transgenic lines were similar, suggesting that enhanced P uptake under soil acidity was indeed largely achieved as an indirect effect of *TaALMT1* enhancing Al resistance. It should be noted that clay acidic soils generally have a strong buffering capacity and, although liming can be used to reduce Al^3+^ in the topsoil, neutralization of subsoil Al^3+^ is often difficult to achieve. In the absence of liming, Al resistance can have an important indirect effect on crop performance *via* both enhanced root proliferation in the topsoil, where P is primarily located on acidic soils (Lynch and Brown, 2001), and improved water acquisition by better root development in the subsoil. With liming, Al tolerance may most strongly benefit crop yields by enhanced water acquisition from deeper, acidic soils layers.

Allelic variation at the sorghum chromosome 3 Al resistance locus, *Alt_SB_* (Magalhaes et al., 2004), where the citrate transporter, *SbMATE*, resides (Magalhaes et al., 2007), explains a large portion of the sorghum Al resistance phenotype. Recently, a sorghum recombinant inbred line (RIL) population was assessed for Al resistance both in lab-based hydroponics (relative root growth) and in the field (grain yield) under +/-Al exposure, in a phenotyping site located at the Embrapa Maize and Sorghum station in Brazil (Carvalho et al., 2016). In that study, sorghum hybrids were also constructed that were either homozygous for the Al-sensitive or -resistant *SbMATE* allele, or heterozygous for *SbMATE*. These hybrids were isogenic, so that *Alt_SB_* alleles from different donors could be compared within a homogeneous genetic background, thus isolating the effect of *SbMATE* from genetic background effects.

The resulting isogenic hybrids were assessed for grain yield in the field on control (absence of Al toxicity in the soil) or in an Al toxic soil with 56% Al saturation in the top soil (0–20 cm) and ∼70% Al saturation in the sub-soil (20–40 cm). A major QTL underlying both Al resistance assessed in hydroponics and grain yield under Al toxicity in the field was co-located with *SbMATE* on sorghum chromosome 3, and explained a large portion of the genetic variance in the Al toxic but not in the non Al-toxic soil. The allele associated with increased Al resistance was donated by the Al tolerant parent, SC283, and the Al resistance allele did not decrease grain yield in the absence of Al toxicity, indicating that no yield penalty arises from Al-induced citrate release elicited by SbMATE. This genetic approach allowed the authors to estimate a consistent effect of a single Al resistance allele of *SbMATE* as a grain yield increase of ∼0.6 ton ha^-1^, both in the RILs and in hybrid combinations. The rather additive gene action of *SbMATE* in grain yield production indicates that, when in homozygosity, *SbMATE* increases grain yield by more than 1.0–ha^-1^, or more than 50% over the population mean. The Al saturation level in the Al toxic site, 56%, is well above the 20% critical level beyond which sorghum yields are reduced (Gourley, 1987). Therefore, most of the yield advantage of *SbMATE* is likely caused by its effect on Al resistance itself. However, the typical acidic soil in question also has high P fixation capacity and P diffusion is known to be highly depend on the soil water content (Novais and Smith, 1999). Therefore, as Al stress and low P availability in general co-exist on acidic soils, a smaller portion of the yield advantage caused by *SbMATE* may have originated from citrate-based enhanced P mobilization (Drouillon and Merckx, 2003) from the soil clays into the root surface, which is expected to favor P uptake.

A more compelling evidence for a pleiotropic effect of SbMATE on P acquisition comes from a genome-wide association mapping study conducted in West Africa (Leiser et al., 2014), which included gene-specific markers developed for *SbMATE* (Caniato et al., 2014). This study revealed that *SbMATE* SNPs were highly associated with grain yield and the associations were found especially under low P conditions for sorghum cultivated in soils at 29 different sites in West Africa, explaining up to 16% of the genotypic variance (Leiser et al., 2014). The average Al saturation was only 10% in the 16 field trials that were analyzed for Al saturation in the Leiser et al. (2014) study, and only one site had Al saturation reasonable above (27.5%) the critical level of Al saturation determined for sorghum (20%, Gourley, 1987). This suggests a direct pleiotropic effect of Al-activated citrated release promoted by SbMATE in enhancing P uptake and sorghum yields under low P availability in West Africa. It should be noted, however, that Al toxicity varies according to the chemical and mineral nature of the soils, which ultimately controls free Al^3+^ activity in the soil solution. Therefore, in sandy soils, such as those commonly found in West Africa, we cannot rule out that higher Al^3+^ activity in some of the sites may have led SbMATE activity to improve sorghum grain yield *via* Al resistance.

### Evidence for a Pleiotropic Role of the STOP1/ALMT1 Module and ALS3 on P Acquisition *via* Changes in Root Morphology in Response to P Deficiency

Recent research findings exposed a possible direct link between AtALMT1 function and both Al resistance and changes in root growth triggered by response to low P (Balzergue et al., 2017; Mora-Macías et al., 2017). Previously, an antagonistic connection was established between phosphate and Fe availability, leading to adjustments in root growth (Müller et al., 2015). It was found that the *LPR1* (ferroxidase)/*PDR2* (P5-type ATPase) module enhances cell-specific Fe and callose deposition in the meristem and elongation zones under low P conditions. Under low Pi, accumulated ROS, possibly resulting from Fe toxicity triggered by Fe^3+^ accumulation in the apoplast *via* LPR1-dependent Fe oxidation, may lead to callose deposition. In turn, according to the proposed model, callose deposition in the RAM under low P impairs cell-to-cell movement of the SHORT-ROOT (SHR) transcription factor, which is important for stem cell maintenance, hence providing a checkpoint for primary root growth control in response to low P.

A mutation screen in Arabidopsis indicated that both ALMT1 and its transcriptional regulator, STOP1, repress primary root growth under -P conditions (Mora-Macías et al., 2017). Furthermore, P deficiency was also shown to upregulate *ALMT1* expression in Arabidopsis, and experiments where exogenous malate was applied to the RAM restored the short root phenotype in *almt1* and *stop1* mutants in a concentration-dependent manner. Fe accumulation in the RAM was found to be required to activate the inhibition of primary root growth under -P conditions (Müller et al., 2015). Hence, the primary root growth inhibition by malate was suggested to occur *via* malate chelating and solubilizing Fe in the rhizosphere, which would promote Fe accumulation in the RAM apoplast (Mora-Macías et al., 2017). Accordingly, the resulting RAM exhaustion process leading to inhibition of the primary root growth under low P (Sánchez-Calderón et al., 2005) happens in the presence of Fe in the growth medium. Callose deposition, which is stimulated by ROS, may be involved in the root elongation inhibition following the model proposed by Müller et al. (2015). Hence, impaired cell-to-cell movement of the SHR transcription factor, which is important for stem cell maintenance, was suggested to lead to meristem exhaustion, inhibiting primary root growth (Müller et al., 2015; Mora-Macías et al., 2017). Because the enhanced proliferation of lateral roots coincides with the inhibition of the primary root (release of root apical dominance) under low P conditions (Sánchez-Calderón et al., 2005), ALMT1 may ultimately increase P uptake on acidic soils *via* increases in total root surface area, thereby favoring P diffusion toward the root surface.

A strikingly similar mechanism for an Al resistance gene leading to changes in root growth as a response to P deficiency has been proposed for ALS3 (Larsen et al., 1996, 2005) and AtSTAR1 (Huang et al., 2010; Belal et al., 2015; Dong et al., 2017). Together, STAR1 and STAR2 (a rice homolog of *als3*) form an ABC transporter implicated in Al resistance likely *via* the transport of UDP glucose into the root apoplast, which is believed to modify the cell wall leading to Al resistance (Huang et al., 2009) as previously discussed in Section “Al Resistance Transporters That do not Transport Organic Acids: Aluminum-Sensitive 3 (ALS3).” The commonality between the putative pleiotropic pathways mediated by *ALMT1* and *ALS3*/*AtSTAR1* is striking, particularly taking into consideration that those genes underlie distinctly different Al resistance mechanisms. Both pathways involve cross-talk between low P responses and Fe homeostasis, with involvement of LOW PHOSPHATE ROOT (LPR) oxidases; mutations in *LPR* leads to reduced Fe^3+^ accumulation in roots and thereby root growth insensitivity to low Pi (Müller et al., 2015; Dong et al., 2017; Mora-Macías et al., 2017). However, the ALS3 pathway involves UDP glucose, which reverses Fe^3+^ overaccumulation and rescues the short root phenotype in *als3* subjected to -P conditions (Dong et al., 2017). However, unlike the T-DNA mutants for *AtALMT1* and *STOP1, als3* shows enhanced inhibition of primary root growth under P deficiency (Dong et al., 2017), suggesting possible antagonism between Al resistance conferred by ALS3 and P acquisition.

These studies offer a radically different stance on root OA release enhancing resistance to low P solely *via* increased P availability in the rhizosphere, as root developmental changes caused by ALMT1/STOP1 and ALS3 appear to be a low P-specific response that is focused on root development. A common physiological basis centered on Fe homeostasis underlying the effect of distinctly different Al resistance pathways encoded by ALMT1/STOP1 and ALS3 on root remodeling under low P seems likely. Should those responses prove to persist for crops cultivated on acidic soils, it will be tempting to speculate that the close soil chemistry associations between Al toxicity and low P availability, which is centered on the presence of Fe and Al oxides, may have resulted in co-selective pressure for pleiotropic mechanisms enabling plants both to tolerate Al^3+^ and to acquire P more efficiently. Nevertheless, there is a strong need for strategies to validate whether the direction of this hypothetical pleiotropic effect is consistent with a positive net benefit on acidic soil performance.

### Are Wall-Associated Kinases Associated With a Joint Effect on Al Resistance and P Acquisition?

Wall-associated kinases (WAKs), which are receptor-like kinase proteins (Kohorn and Kohorn, 2012) that span the plasma membrane and extend out into the cell wall (He et al., 1999), have been shown to play roles in cell expansion, development, morphogenesis, and defense responses to environmental stimuli (Sivaguru et al., 2003; Brutus et al., 2010; Kohorn and Kohorn, 2012; Gramegna et al., 2016; Mangeon et al., 2016). Sivaguru et al. (2003) reported that *AtWAK1* expression was rapidly induced by Al and disappeared after 9 h of Al exposure and that transgenic plants overexpressing *AtWAK1* showed enhanced Al resistance. Recently, a T-DNA knockout of the glycine-rich protein, AtGRP3, which interacts with AtWAK1 (Park et al., 2001), has also been shown to enhance Al resistance in Arabidopsis, similar to *AtWAK1* (Mangeon et al., 2016). However, *AtGRP3* expression was not modulated by Al and *grp3* had a long root phenotype in the absence of Al exposure. Therefore, it remains to be verified whether the lower root growth inhibition in *grp3* exposed to Al compared to the wt is in fact due to a mechanism enhancing Al resistance or is influenced to some extent by a leaky *grp* mutation, based on the role for *AtGRP3* in repressing root growth.

Wall-associated kinases form a subfamily within the receptor kinase (RLKs)/Pelle superfamily, which includes other subfamilies such as WAK-like kinase (WAKL) and Leaf rust 10 disease-resistance locus receptor-like protein kinase (LRK10), that share similar protein architectures with the WAK proteins (Shiu and Bleecker, 2003; Hou et al., 2005; Lim et al., 2014). The general WAK protein architecture features an extracellular moiety containing a cysteine-rich (Cys-rich) galacturonan-binding domain (Gub_Wak), epidermal growth factor (EGF) repeats, and a TM domain, in addition to a cytoplasmic serine/threonine kinase domain (Anderson et al., 2001; Decreux and Messiaen, 2005; Decreux et al., 2006).

Using association mapping, Hufnagel et al. (2014) showed that sorghum homologs of the rice serine/threonine receptor kinase, OsPSTOL1 (Gamuyao et al., 2012), are involved in increases in root surface area leading to enhanced P acquisition and grain yield under low P availability in the soil. In sorghum, these SbPSTOL1 proteins are predicted to have a signal peptide consistent with the targeting to a secretory pathway, as well as a TM domain and cell wall association domains. For example, the Sb03g006765 protein associated with P efficiency and increased root surface area is predicted to have a Cys-rich GUB_Wak domain and a wall-associated receptor kinase domain (WAK_association) located C-terminal to the GUB_Wak domain. Similarities between SbPSTOL1 and WAK proteins such as AtWAK1, which appears to be involved in Al resistance (Sivaguru et al., 2003), arise primarily from the presence of the GUB_Wak and TM domains, similar intron–exon organization, and a genomic localization in tight physical clusters (Hufnagel et al., 2014). Recent studies have suggested that amino acids in the Gub_Wak domain bind covalently to native pectins and oligogalacturonides in the cell wall (Verica and He, 2002; Decreux and Messiaen, 2005; Decreux et al., 2006; Kohorn and Kohorn, 2012; Kohorn et al., 2016). This leads us to speculate that SbPSTOL1 proteins may function as WAKs, functioning as receptors for the activation of signaling cascades in response to extracellular stimuli (in this case, P deficiency). However, in place of the EGF repeats, which is a hallmark of WAK proteins (Kanneganti and Gupta, 2008), WAKL and LRK10 members, including Sb03g006765, possess a WAK_association domain.

**FIGURE 1:**
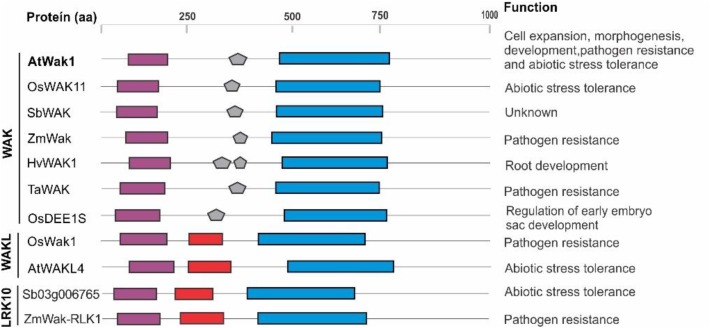
Structure and possible function of WAK, WAKL, and LRK10 members of the RLKs/Pelle superfamily. Protein domains are represented with the following colors: purple (Gub_Wak), red (Wak association), and blue (Kinase). Amino acidic sequences were obtained from the National Center for Biotechnology Information (NCBI; http://www.ncbi.nlm.nih.gov) and Phytozome (www.phytozome.org) databases. The SbWAK protein sequence is available at Phytozome under accession Sobic.004G008100.1. The amino acidic alignment was performed with ClustalW (www.ebi.ac.uk/Tools/msa/clustalw2), and protein domain prediction was carried out using the Pfam (http://pfam.xfam.org/) and Smart (http://smart.embl-heidelberg.de/) tools. Inferences on functions for AtWak1(Anderson et al., 2001; Sivaguru et al., 2003; Brutus et al., 2010; Gramegna et al., 2016), OsWAK11(Hu et al., 2014), ZmWAK (Zuo et al., 2015), HvWAK1 (Kaur et al., 2013), TaWAK (Yang et al., 2014), OsDEE1S (Wang et al., 2012), OsWak1(Li et al., 2009), AtWAKL4 (Hou et al., 2005), Sb03g006765 (Hufnagel et al., 2014), and ZmWak-RLK1 (Hurni et al., 2015) are shown.

The GUB_Wak domain is present in certain plant proteins suggested to be involved in responses to abiotic and biotic stresses that belong to three subfamilies in the RLK superfamily, the WAKL, WAK, and LRK10 subfamilies (with Sb03g006765 within the LRK10 subfamily). These proteins are depicted in **Figure 1**. Sequence alignment of the GUB_Wak amino acidic sequences in these proteins does not show a high degree of conservation. However, this domain has conserved clusters of hydrophobicity that are essential for the association of these proteins *via* the extracellular residues, including a Cys-rich region and a conserved YPF motif. Therefore, it remains to be seen whether the SbPSTOL1 proteins functionally work as WAKs such as AtWAK1. If so, given the predicted role for SbPSTOL1 in enhancing root growth and P uptake in sorghum, this class of proteins could jointly control Al resistance and P uptake.

## Conclusion

We are at a stage in research on crop plant adaptation to acidic soils where a number of different Al resistance genes have been identified. These genes have been discovered using a variety of both forward and reverse genetic strategies, ranging from candidate genes validated primarily *via* ectopic overexpression in transgenic plants or identified *via* mutant screens to map-based cloning of Al resistance genes underlying loci previously known to play a role in the genetic variation of Al resistance. In most cases, very little work has been done to translate the findings from the basic research used to identify and characterize the genes to practical applications to generate crop varieties in breeding programs. The research that connects with genetic variation present within crop species to identify Al resistance genes is certainly the most amenable to providing molecular tools for the breeding of crops with improved production on acidic soils. In the cases where genetic determinants of Al resistance have been found by other approaches, efforts to assess whether those determinants are also active in crop plants in field conditions are sorely needed if the ultimate goal is indeed to generate crops more adapted to cultivation on acidic soils. While the effect of Al resistance on crop performance on acidic soils is known, pleiotropic effects of such genes on P uptake efficiency needs to be explored in crop species grown in the field. In both cases, detailed quantification is needed to gage the true potential of Al resistance genes in coping with agriculture in stress-prone areas. Particularly in a scenario where global climate change is resulting in greater drought stress, the potential of those genes to ensure food security worldwide may be far greater than initially believed.

## Author Contributions

JM and MP delineated and wrote this review. LM and LK wrote this review. LK also edited the manuscript.

## Conflict of Interest Statement

The authors declare that the research was conducted in the absence of any commercial or financial relationships that could be construed as a potential conflict of interest. The reviewer MD and handling Editor declared their shared affiliation.
